# Application of Biotechnology in Myocardial Regeneration-Tissue Engineering Triad: Cells, Scaffolds, and Signaling Molecules

**DOI:** 10.1155/2013/236893

**Published:** 2013-02-17

**Authors:** Daria Nurzynska, Maria-Elena Padin Iruegas, Clotilde Castaldo, Patrick Müller-Best, Franca Di Meglio

**Affiliations:** ^1^Department of Public Health, University of Naples “Federico II”, 80131 Naples, Italy; ^2^Translational Oncology Laboratory (Clinical Hospital Santiago de Compostela), Department of Functional Biology and Health Sciences, University of Vigo, 36310 Vigo, Spain; ^3^Department of Internal Medicine, Marienhaus Hospital, Saarland, 66564 Ottweiler, Germany

Following the recent advances in cardiac stem cell biology, cardiac tissue has become one of the targets of regenerative medicine. With the array of approaches that are currently being tested and the questions that they raise, it becomes increasingly clear that the regeneration of adult myocardium will require the integration of numerous factors that govern cardiac tissue biology in normal and pathological conditions. The current challenge in myocardial tissue engineering and regeneration is to establish a perfect combination of three components: cells, scaffolds, and signaling molecules [1].

Since the recognition that the mammalian heart is characterized by cardiomyocyte turnover throughout the lifespan of an individual, a number of different cardiac stem cell populations have been identified in the postnatal and adult hearts. A desirable candidate cell for cardiac tissue engineering and regeneration would be autologous, capable of differentiation into all the cardiac cell types and of functional integration, and resistant to malignant transformation. The preclinical studies and clinical trials have employed different cell types, including embryonic stem cell-derived and fetal cardiomyocytes, skeletal myoblasts, bone marrow or peripheral blood-derived endothelial progenitors, haematopoietic and mesenchymal stem cells, and recently, reprogrammed adult somatic cells (induced pluripotent stem cells). These multiple cell choices, apart from being accompanied by important ethical and safety concerns, led to a rather uniform outcome, which consisted of a low degree of improvement in cardiac contractile function. Moreover, a regenerated tissue band, which would integrate functionally with the surviving myocardium, was observed only in few cases [2].

The choice of cell population for myocardial regeneration should be based on the knowledge of stem cell biology and the mechanisms that regulate and control cardiac progenitor proliferation, apoptosis, and differentiation in the normal and pathological conditions [3]. Recently, short noncoding RNAs, named micro-RNAs (miRs), have been found to control gene expression by blocking mRNA translation and inducing its degradation. MiR-1 was among the first in this group that had been identified as regulators of muscle cell proliferation and differentiation [4]. In this special issue, F. Huang et al. demonstrated that the downstream molecular target of Notch, Hes-1, decreased, while the expression of specific cardiac genes increased, in mesenchymal stem cells infected with lentiviral vectors carrying miR-1. This miR may be useful for stimulation of cell fate decision towards cardiomyocyte lineage.

The group of M. E. Roehrich et al. identified by flow cytometry yet another stem cell population, with high expression and activity of aldehyde dehydrogenase (ALDH), in the adult heart, and the results of the characterization of these cells are published for the first time in the present special issue. This population is enriched for cells expressing Sca-1, CD34, CD90, CD44, and CD106, which indicates that it is a heterogeneous cell population, and presents mesenchymal stem cell-like differentiation potential. Interestingly, ALDH bright cells had been already sorted from human cord blood, bone marrow and peripheral blood and identified in nonhaematopoietic tissues as neural, muscle, colonic, and mammary stem cells. The activity of this enzyme allows cells to survive oxidative stress and can influence numerous functions that are crucial for stem cell survival in hypoxic environment. Indeed, cells with high activity of ALDH homed to ischemic sites (in the infarcted heart and hind limb ischemia model) and mediated local vasculogenesis [5]. Recent preliminary results indicate that treatment with ALDH bright autologous bone marrow cells is safe and may provide functional benefits in patients with chronic myocardial ischemia [6]. However, cell number and method of delivery may influence the efficacy of ALDH bright and all other cell populations used for myocardial regeneration.

Many factors determine the outcome of regenerative cardiovascular therapy and are the possible reasons behind the variable results. In fact, the methods for cell isolation and purification, the procedures for their storage or propagation, the phenotype and number of injected cells, and the methods of cell administration are hugely inconsistent between studies. The current stem cell delivery methods for myocardial repair are reviewed in the present special issue by C. C. Sheng et al. The possibilities are continuously expanding and the report of the International Society for Cardiovascular Translational Research, with the recommendations for the delivery of stem cells that would be comparable between groups [7], should be soon updated to include also bioengineered cardiac tissue transplantation.

An ideal scaffold for tissue engineering must be biocompatible, allow cell growth and differentiation, and integrate (or degrade in time-controlled manner) into the host tissue. While patch-based scaffolds for cardiac regeneration must reach the dimensions relative to infarct size in humans, comply with the tension developed in the ventricular wall during systole and diastole cycles, and still be applicable through minimally invasive procedures, injectable scaffolds must be able to pass through a small gauge catheter and reconstitute their fibrous structure through gelation. Preclinical studies indicate that the combined cell-biomaterial scaffold therapy is superior to cell therapy alone [8], but the evaluation of cellular effects *in vitro* would be even more reliable and easier to translate in clinical application if performed in 3D culture platforms. 3D environment and high cell density play an important role in supporting the phenotypes of cells of cardiac lineages. Many of these aspects of scaffold composition and cellular biology are discussed in this special issue.

First, the original contribution of K. Vukusic et al. describes the behaviour of cardiomyocytes, endothelial cells, fibroblasts, and c-kit-positive cardiac primitive cells in high density sphere cultures. The simple method of sphere formation and culture, starting from the pellet of cardiac cells that rounded up and formed spherical structure that floated in the culture media, adds to the previously characterized methods of cardiosphere generation (hanging drop or standard plate suspension culture). Growth of cardiac cells as cardiospheres enhanced expression of stem cell-relevant markers. Next, L. Ikonen et al. examined collagen-like synthetic self-assembling nanofiber hydrogels for their suitability for neonatal rat cardiomyocyte and human embryonic stem cell-derived cardiomyocyte culture in 2D. Although the differentiation of stem cells into cells of cardiac lineages in these hydrogels would be more indicative of their application in myocardial regeneration, it is of note that hydrogel was able to maintain the beating ability of differentiated cardiomyocytes *in vitro*. As such, it can represent a useful model for preclinical testing of cellular populations and stem cell-derived cardiomyocytes for tissue engineering. In addition, the research of D. Avitabile et al. offers the basis for a further optimization of stem cell and scaffold processing for myocardium repair, showing that embedding human cord blood-derived CD34+ cells into a collagen I-based hydrogel containing cytokines is a suitable strategy to promote proliferation and protect these cells from hypoxia-induced apoptosis. The properties of these injectable composites make them promising candidates for myocardial tissue regeneration and warrant further studies on their potential therapeutic application. 

With these and other scientific advances in basic science research, which provide clinicians with the tools for replacing damaged myocardium, the concept of cardiac regeneration should hopefully progress to a level that will allow the translation and application of tissue engineering triad, that is, cells, scaffolds, and signalling molecules, in the clinical setting. The success of this integrated approach will depend on the carefully designed and analyzed experiments and clinical trials.

*Daria Nurzynska* *Maria-Elena Padin Iruegas* *Clotilde Castaldo* *Patrick Müller-Best* *Franca Di Meglio*

## References

[1] Lakshmanan R, Krishnan UM, Sethuraman S (2012). Living cardiac patch: the elixir for cardiac regeneration. *Expert Opinion on Biological Therapy*.

[2] Nurzynska D, Castaldo C, Montagnani S, Di Meglio F, Atala A (2012). Cardiac progenitor and stem cell biology and therapy. *Progenitor and Stem Cell Technologies and Therapies*.

[3] Nurzynska D, Di Meglio F, Romano V (2013). Cardiac primitive cells become committed to a cardiac fate in adult human heart with chronic ischemic disease but fail to acquire mature phenotype—genetic and phenotypic study. *Basic Research in Cardiology*.

[4] Heinrich EM, Dimmeler S (2012). MicroRNAs and stem cells: control of pluripotency, reprogramming, and lineage commitment. *Circulation Research*.

[5] Balber AE (2011). Concise review: aldehyde dehydrogenase bright stem and progenitor cell populations from normal tissues: characteristics, activities, and emerging uses in regenerative medicine. *Stem Cells*.

[6] Perin EC, Silva GV, Zheng Y (2012). Randomized, double-blind pilot study of transendocardial injection of autologous aldehyde dehydrogenase-bright stem cells in patients with ischemic heart failure. *American Heart Journal*.

[7] Dib N, Menasche P, Bartunek JJ (2010). Recommendations for successful training on methods of delivery of biologics for cardiac regeneration: a report of the International Society for Cardiovascular Translational Research. *Cardiovascular Interventions*.

[8] Sarig U, MacHluf M (2011). Engineering cell platforms for myocardial regeneration. *Expert Opinion on Biological Therapy*.

